# Perspectives on a peer-driven intervention to promote pre-exposure prophylaxis (PrEP) uptake among men who have sex with men in southern New England: a qualitative study

**DOI:** 10.1186/s12913-024-11461-7

**Published:** 2024-09-04

**Authors:** Jun Tao, Hannah Parent, Ishu Karki, Harrison Martin, Sarah Alexandra Marshall, Jhanavi Kapadia, Amy S. Nunn, Brandon D. L. Marshall, Henry F. Raymond, Leandro Mena, Philip A. Chan

**Affiliations:** 1 Division of Infectious Diseases, Brown University Warren Alpert Medical School, 593 Eddy Street, Providence, RI 02903 USA; 2 Division of Infectious Diseases, The Miriam Hospital, 11 4th street, Providence, RI 02906 USA; 3 Department of Epidemiology, Brown University School of Public Health, 121 South Main Street, Providence, RI 02912 USA; 4 Department of Health Behavior & Health Education, Fay W. Boozman, College of Public Health, University of Arkansas for Medical Sciences, Little Rock, AR USA; 5 College of Osteopathic Medicine, University of New England, 11 Hills Beach, 04005 Biddeford, Maine, USA; 6 Department of Behavioral and Social Sciences, Brown University School of Public Health, Providence, RI USA; 7 Department of Biostatistics and Epidemiology, Rutgers School of Public Health, Piscataway, NJ 00854 USA; 8 Division of STD Prevention, Centers for Disease Control and Prevention, Atlanta, GA 30329 USA

**Keywords:** Peer-driven intervention, Men who have sex with men (MSM), HIV pre-exposure prophylaxis (PrEP), Black/African American, Hispanic/Latino

## Abstract

**Background:**

Pre-exposure prophylaxis (PrEP) is a highly effective pharmaceutical intervention that prevents HIV infection, but PrEP uptake across the US has been slow among men who have sex with men (MSM), especially among Black/African American (B/AA) and Hispanic /Latino (H/L) MSM. This study investigates the acceptability and essential components of a peer-driven intervention (PDI) for promoting PrEP uptake among MSM, with a specific focus on B/AA and H/L communities.

**Methods:**

We conducted 28 semi-structured, qualitative interviews with MSM in southern New England to explore the components of a PDI, including attitudes, content, and effective communication methods. A purposive sampling strategy was used to recruit diverse participants who reflect the communities with the highest burden of HIV infection.

**Results:**

Of 28 study participants, the median age was 28 years (interquartile range [IQR]: 25, 35). The sample comprised B/AA (39%, *n* = 11) and H/L (50%, *n* = 14) individuals. Notably, nearly half of the participants (46%) were current PrEP users. We found that many participants were in favor of using a PDI approach for promoting PrEP. Additionally, several participants showed interest in becoming peer educators themselves. They emphasized the need for strong communication skills to effectively teach others about PrEP. Moreover, participants noted that peer education should cover key topics like how PrEP works, how effective it is, and any possible side effects.

**Conclusions:**

Our study shows that effective PDIs, facilitated by well-trained peers knowledgeable about PrEP, could enhance PrEP uptake among MSM, addressing health disparities and potentially reducing HIV transmission in B/AA and H/L communities.

**Supplementary Information:**

The online version contains supplementary material available at 10.1186/s12913-024-11461-7.

## Introduction

In 2021, gay, bisexual, and other men who have sex with men (MSM) accounted for 67% of all new HIV infections in the United States (US) [1]. Black/African American (B/AA) MSM are the most affected subpopulation, followed by Hispanic/Latino (H/L) MSM [1]. B/AA and H/L MSM each represent less than 1% of the population [2], but account for 25% and 22% of all new HIV diagnoses in 2021, respectively [1]. Given this concentrated epidemic, the *2025 National HIV/AIDS Strategy* lists MSM as one of the high-priority populations for HIV initiatives, in particular Black, Latino, and American Indian/Alaska Native men, as focusing resources on these populations would reduce HIV incidence disparities among MSM and achieve greater impact in reducing HIV incidence overall [3].

Pre-exposure prophylaxis (PrEP) — a highly effective pharmaceutical intervention against new HIV infection [4–6]— has the potential to dramatically reduce HIV incidence among MSM in the US [7, 8]. Despite the proven efficacy and recommendation by the Centers for Disease Control and Prevention (CDC) that all sexually active persons at risk of HIV acquisition could benefit from PrEP, PrEP uptake has been slow across the US, especially among B/AA and H/L MSM [9–11]. In 2021, B/AA individuals comprised 14%, H/L individuals made up 17%, while White individuals accounted for 65% of all persons prescribed PrEP in the US [12]. Other studies have found that B/AA and H/L MSM who initiate PrEP are significantly less likely to be retained in care at three months relative to white MSM [13, 14]. B/AA and H/L MSM are also more likely to have limited awareness about PrEP [15], low perceived HIV risk [16], medical mistrust [17], and experience stigma [18] and financial burdens [19]; all of which contribute to suboptimal PrEP uptake and retention in PrEP care [20–25].

Community-based outreach approaches and peer-driven interventions (PDI) have the potential to mitigate these barriers and enhance PrEP uptake among MSM, especially B/AA and H/L MSM. PDIs involve recruiting peer educators and then encouraging them to educate and motivate members of their social network(s) for PrEP uptake. Research demonstrates that PDIs are both cost-effective for engaging hard-to-reach populations [26, 27], and efficient for disseminating HIV education, promoting condom use, and expanding HIV testing among MSM [28, 29]. However, there is limited research on the feasibility of a PDI for PrEP promotion, apart from a pilot study conducted by the authors from 2018 to 2019 [30]. Most of the 15 participants in the pilot study viewed positively a PDI to promote PrEP. The current study investigated the acceptability and effectiveness of PDIs for PrEP uptake among MSM, focusing on B/AA and H/L subgroups. Acknowledging their distinct needs, the study aimed to identify PDI components tailored to each group, addressing specific barriers in PrEP adoption. Findings from this study can inform the development and implementation of future PDIs for PrEP promotion in the US.

## Methods

### Study population and recruitment

Our research staff conducted 28 in-depth interviews with MSM between October 6th, 2020, and September 2nd, 2022. Participants were recruited from multiple venues, including clinical outreach at The Miriam Hospital (TMH) Immunology Center; lesbian, gay, bisexual, transgender, and LGBTQ + bars and community-based organizations in Providence, Rhode Island; and LGBTQ + email-based listservs in southern New England (Rhode Island, Connecticut, and Massachusetts). Participants were eligible if they: (a) were 18 years of age or older, (b) were assigned male at birth and identified as a man, (c) had sex with men in the past three months, and (d) had a HIV negative status. Our sampling approach was purposive, aiming to encompass a wide range of experiences among B/AA and H/L MSM with different PrEP statuses, including both current users and non-users [31–33].

### Interviews and data collection

Twenty-eight eligible study participants were invited to participate in a 45–60-minute one-on-one interview with a trained researcher. Study staff decided to conduct all interviews via Zoom, a HIPPA-compliant online meeting platform, due to the coronavirus disease 2019 (COVID-19) pandemic. Interviewers referred to an interview guide that they developed specifically for the purpose of the current study (Supplementary File 1). Interview questions covered the following topics: (1) awareness and acceptability of PrEP; (2) social network characteristics (including both physical and virtual interactions) and acceptability of promoting PrEP through these networks; (3) facilitators and barriers to PrEP uptake; (4) potential peer-delivered intervention components and related content to support PrEP initiation; (5) characteristics of ideal PrEP educators (e.g., leadership, responsibility, and passion and commitment to HIV prevention); and (6) cultural nuances about PrEP. As PDIs are based on existing social networks to reach individuals who may be at high risk of HIV infection, it is crucial to understand the dynamics of these networks and provide insights into how individuals are connected both directly and indirectly. Enrollment ended when preliminary data analysis reflected thematic saturation [34]. Specifically, we continued enrolling participants until data saturation was achieved, a point at which additional interviews ceased to yield novel insights or significantly alter our understanding of the research topic. Each participant was compensated $50 for their time. The Institutional Review Board at The Miriam Hospital approved the study (IRB number:1594759).

### Data analysis

Interviews were digitally recorded and then transcribed verbatim by an external HIPAA-certified transcription company. Following transcription, research staff meticulously reviewed the transcripts to ensure they accurately reflected the recorded content, paying close attention to linguistic nuances and context-specific details. In addition to conducting interviews, research staff took notes, completed standardized debriefing forms immediately following the interview, and reviewed these forms with study investigators during weekly team meetings. Qualitative data were analyzed iteratively during data collection by a trained research assistant, and the interview guides were adapted as needed to address any unanticipated, emergent themes. The primary themes were organized and distilled into the primary findings presented here. This method allowed us to determine when we reached saturation in data collection. Qualitative and mixed methods data analysis was conducted using Dedoose, a versatile software platform designed for analyzing qualitative and mixed methods research data. Dedoose facilitates the organization, coding, and interpretation of complex datasets, enhancing the efficiency and depth of analysis [35]. The study team developed a coding scheme based on the interview protocol and transcripts of 28 interviews. After an initial round of coding, the research team met to discuss the coding and revise the codebook. Three independent qualitative analysts coded the transcripts. Discrepancies in the coding between any two analysts were resolved by the third analyst who assigned the final code. We used a thematic analysis strategy to analyze the data [36]. The research team reviewed and analyzed data to identify themes within the domains from the interview guide.

## Results

### Demographic characteristics

Of the 28 study participants, the median age was 28 years old (interquartile range [IQR]: 25, 35). The majority reported having a college education or above (78%), having health insurance (93%), identifying same-sex sexual orientation (79%), and being single (57%). B/AA and H/L individuals accounted for 39% (*N* = 11) and 50% (*N* = 14) of all participants, respectively. Thirteen individuals (46%) reported currently taking PrEP; five individuals (18%) reported previous PrEP use; nine individuals (32%) had never taken PrEP before; and one individual (4%) had never heard of PrEP (Table 1).

**Table 1:** Demographics by PrEP status

	Not on PrEP	On PrEP	Total	*P*-value
	N = 15	N = 13	N = 28	
Age	33 (25–36)	27 (24–32)	28 (25–35)	0.27
Race				
*White*	7 (47%)	4 (31%)	11 (39%)	0.060
*Black*	3 (20%)	8 (62%)	11 (39%)	
*Other*	5 (33%)	1 (8%)	6 (22%)	
Ethnicity				
*Not Hispanic/Latino*	7 (47%)	7 (54%)	14 (50%)	0.71
*Hispanic/Latino*	8 (53%)	6 (46%)	14 (50%)	
Sexual orientation				0.84
*Only same-sex*	12 (80%)	10 (77%)	22 (79%)	
*Bisexual*	3 (20%)	3 (23%)	6 (21%)	
Relationship status				
*Single*	7 (47%)	9 (69%)	16 (57%)	0.49
*Monogamous*	4 (27%)	2 (15%)	6 (21%)	
*Non-monogamous*	4 (27%)	2 (15%)	6 (21%)	
Education				
*High school or less*	1 (7%)	1 (8%)	2 (7%)	0.18
*Some college*	4 (27%)	0 (0%)	4 (14%)	
*College*	3 (20%)	6 (46%)	9 (32%)	
*Graduate*	7 (47%)	6 (46%)	13 (46%)	
Student status				
*No*	10 (67%)	9 (69%)	19 (68%)	0.89
*Yes*	5 (33%)	4 (31%)	9 (32%)	
Employment				0.45
*Full-time*	10 (67%)	11 (85%)	21 (75%)	
*Part-time*	4 (27%)	2 (15%)	6 (21%)	
*Unemployed/disability*	1 (7%)	0 (0%)	1 (4%)	
Income				
*Less than $25,000*	4 (27%)	2 (15%)	6 (21%)	0.49
*$25,000-$75,000*	7 (47%)	9 (69%)	16 (57%)	
*More than $75,000*	4 (27%)	2 (15%)	6 (21%)	
Insurance				0.16
*None*	2 (13%)	0 (0%)	2 (7%)	
*Private*	8 (53%)	11 (85%)	19 (68%)	
*Medicare or Medicaid*	5 (33%)	2 (15%)	7 (25%)	

### Themes identified during interviews

Thematic analysis suggested several key themes for informing the development and implementation of a PDI to promote PrEP uptake among MSM, especially B/AA and H/L individuals. Table 2 presents a list of the themes generated from participant interviews, including: (1) characteristics of social networks; (2) the role of peers in increasing PrEP awareness and knowledge; (3) attitudes towards a PrEP PDI; (4) ideal characteristics of PrEP peer educators; (5) key components of PrEP education; (6) suggested approaches for initiating conversations about PrEP; (7) cultural barriers to initiating PrEP; (8) barriers to initiating PrEP for young adults; (9) suggestions for PrEP education content for peer educators; and (10) the impact of COVID-19 on social and sexual behaviors. These themes are described below along with illustrative quotes.

**Table 2:** Descriptions of identified themes

Themes	Description
Diversity of social networks	- Most participants reported having a racially/ethnically diverse social network, consisting of childhood friends, and friends made via college, work, or mutual friend - Most participants reported having several friends who shared their MSM identity
Role of peers in increasing PrEP awareness and knowledge	- Seven out of 28 participants reported first hearing about PrEP from their peers, which motivated them to learn more about PrEP - Eight out of the thirteen participants who were currently taking PrEP reported already playing the role of a PrEP peer educator among their social groups (including straight friends, friends who are MSM, and sexual partners)
Positive attitude towards a PrEP PDI	- Participants unanimously positively endorsed a PDI approach for PrEP promotion - Some participants emphasized the importance of peer educators sharing identities with the communities being targeted by a PrEP PDI intervention - Most participants reported willingness to be a peer educator in their communities to improve PrEP awareness
Ideal characteristics of peer educators	- Most participants agreed that peer educators should have strong communication skills, be friendly and outgoing, respectful, a good listener, truthful, and knowledgeable about PrEP
Critical components of PrEP education	- A few participants highlighted the importance of presenting current HIV data during PrEP education, while also underscoring the high risk of HIV acquisition for MSM - Some participants were unaware of the efficacy of PrEP and expressed wanting to see study data demonstrating PrEP’s high effectiveness in preventing HIV - Participants were also interested in developing a better understanding of how PrEP works and the different PrEP options - Participants thought a PrEP education should emphasize the importance of taking PrEP as prescribed/PrEP adherence, as well as include information about available financial support
Approaches to initiating conversations about PrEP	- A range of approaches were identified by participants, including: letting a conversation about PrEP occur naturally, asking their peers directly if they had heard of PrEP, sharing their personal PrEP-related experiences, providing an informational sheet about PrEP, or only providing information about PrEP if someone asks about it first - Text messages, social media, and in-person meetings were all identified by participants as preferred methods to deliver a PrEP PDI - Some participants suggested text messaging would be the easiest and most convenient delivery method, and that social media would be able to reach a larger audience quickly, while others demonstrated a preference for one-on-one and/or in-person meetings
Cultural barriers to initiating PrEP	- Participants generally agreed that being raised in or belonging to a culture that wasn’t accepting of same-sex relationships, or perceived conversations about sexual health as taboo, could make it difficult to initiate PrEP or even discuss PrEP - Some B/AA and H/L participants shared their own experiences on how their identity, or the community with which they share an identity with, has at times been a barrier in accessing PrEP, initiating PrEP, or discussing PrEP
Unique PrEP uptake barriers for young adults	- One young adult participant mentioned that being on their parents’ insurance was a barrier in initiating PrEP, as it would lead to a difficult conversation with their parents - Another participant (early twenties) shared that their college did not have adequate resources to prescribe them PrEP
Suggestions for intervention components	- Several suggestions were made for a PrEP PDI best practices, such as using the Spanish language alongside English in messages, advertisements, and program materials - Some participants recommended reaching a larger MSM audience at LGBTQ oriented events - A few participants suggested utilizing smartphone apps as both sources of PrEP information, or for PrEP promotion (ex: advertising PrEP on dating apps)
Impact of COVID-19 on social and sexual activities	- All participants reported that COVID-19 changed their social and sexual activities, minimizing their in-person social activities and/or reducing sexual activity - Some participants reported taking a temporary break from PrEP during the pandemic, since their risk of HIV acquisition had decreased due to less sexual encounters and/or fewer sexual partners - Some participants reported being forced to take a temporary break from PrEP during the pandemic due to concerns and barriers related to COVID-19 - At-home delivery of PrEP was a promising method of obtaining PrEP during the pandemic, but a couple of participants reported having major issues with the delivery service that negatively impacted their PrEP adherence

### Social network characteristics

Most participants reported having racially and ethnically diverse social networks. They usually considered less than ten people to be very important in their lives; among those they considered important, they mentioned family members, people with whom they had grown up, or more recent connections made at college, work, or through mutual friends. The majority of participants saw or communicated frequently with two to three individuals.

*“It really is a mix. I have white friends. My best friend is actually Palestinian*,* and*,* yeah*,* Asian friends*,* Mexican friends. It’s a mix…That’s the overall ethnic diagnosis. [Laughter] Well*,* yeah*,* I just meet friends*,* and they come in all flavors.” - Asian/non-Hispanic male*,* mid-twenties*.

Most participants had several friends who self-identified as gay or bisexual men.

*“[I have] quite a few [gay and bisexual friends]. I don’t know in terms of numbers…but I would probably say probably around 20 or 30 people. This is all from goin’ to college and some people from back home. In terms of my close-knit circle of friends that identify as gay and bisexual*,* I would probably say around five or six… Maybe ten max.” - Black/Hispanic male*,* early-twenties*.

### The role of peers in raising PrEP awareness

Of 28 participants, seven reported that they first heard about PrEP from their peers. Their interest in PrEP was sparked by the knowledge and experience their peers shared, motivating them to explore and learn more about PrEP.

*“There was a friend of mine who told me that he was taking it. I asked what it was*,* and he told me… I was like*,* “Oh*,* okay.” Then*,* I looked it up on the internet and that’s when I saw and learned a little more about it.” – Black/non-Hispanic male*,* late-twenties*.

Of thirteen individuals who were currently taking PrEP, eight reported already playing the role of a peer educator within their social network. This included having conversations about PrEP with LGBTQ friends, straight friends, and sexual partners.

*“From my friends’ circle*,* three of my college friends have gone into PrEP after I have talked to them. The three of them identify as African Americans…Every knowledge that I gain that will affect [my friend group]*,* I will try to share that [knowledge with them] as much as I can.” - Black/Hispanic male*,* late-twenties*.

*“I am an advocate for PrEP…I talk to people about it. All of my gay friends I’ve spoken to about it*,* partners that I have hooked up with in the past I’ve spoken to about it…I think it’s something we should all know is available as an option.” – White/Hispanic male*,* mid-thirties*.

### Attitude toward a PDI approach

All participants had a positive attitude toward using a PDI approach for PrEP promotion. Many thought that members of their own communities would be a reliable source for disseminating PrEP knowledge and capable of motivating their peers to seek PrEP counseling and care.

*“I think [a community member] is helpful because you would trust that person*,* so I think it’s important ‘cause—yeah. I think it’s more effective and more impactful than just reading something online.” – Asian/Hispanic male*,* mid-twenties*.

Most interviewees reported that they would be willing to be a peer educator to improve PrEP awareness and reduce rates of HIV infection in their communities.

*“I’d love to take action within the queer community and help support HIV prevention…that would be something I’d be very interested in. Do I have enough education about it? No. But it’s definitely something that I feel like I’d love to learn more about and be able to pass that on.” – White/non-Hispanic male*,* early-twenties*.

### Characteristics of peer educators

Most participants stated that peer educators should have strong communication skills to effectively approach members of their social network for PrEP education. In addition, participants expected PrEP educators to be friendly, outgoing, respectful towards others, and willing to listen. According to participants, PrEP educators should also be able to ensure members in their social network are comfortable, be trustworthy, and highly knowledgeable about HIV and PrEP.

*“I think characteristics they should have is that they should be*,* first of all*,* social. Obviously*,* they have to be able to communicate to people*,* be willing to give people the information*,* be patient with their questions…and treat people with respect when answering the questions.”* - *Black/non-Hispanic male*,* late-twenties*.

*“I think a PrEP promoter should be outgoing*,* engaging. They should also be willing to listen because there are a number of people*,* especially in the African American community*,* who are going to be distrustful of someone who’s pushing a medicine on them. I think that a PrEP promoter should also be willing to hear a ‘no’ and not want to immediately get defensive or get argumentative.”* - *Black/non-Hispanic male*,* mid-thirties*.

*“I think [peer educators] should know the effectiveness of [PrEP]. They should know who is considered high risk and who are the ones that should probably be taking it. Definitely should know what some of the side effects of it can be. I think [a peer educator] should be someone who’s actually taking it themselves*,* because then they have the firsthand—I get that everyone’s body is different*,* but it’s a little bit more comforting hearing it from someone who’s actually taking it. I think that they should just know general things about HIV.” – Black/non-Hispanic male*,* late-twenties*.

### Critical components for PrEP education

Participants identified components that are critical to include when providing PrEP education. The efficacy of PrEP, how it works in the body, and PrEP side effects were common suggestions for PrEP education content. Since there are several forms of PrEP available, including multiple daily oral formulations and long-acting injectable formulations, participants wanted to learn more about these different options. A few participants also mentioned that PrEP education should underscore the high risk of HIV acquisition for MSM, especially B/AA and H/L individuals, as well as emphasize the importance of taking PrEP as prescribed.

*“I think you know the things that you probably wanna cover. It’s like*,* the risk—people knowin’ their risk*,* one; two*,* the options that they can take. There’s Truvada and there’s Descovy. You mentioned those two things. What’s the difference between them and how they both basically benefit the same thing.” – Black/Hispanic male*, *early-twenties*.

*“I should definitely know about both the good and the bad cases of people who have taken it. People who have experienced negative side effects*,* what they were and what the percentage of that is if they have those statistics. Then*,* like I said*,* just the effectiveness of if you take it every day like you’re supposed to.” - Black/non-Hispanic male*,* late-twenties*.

Some participants stated that they would like to learn more information about PrEP’s efficacy and that being equipped with this knowledge would help them feel more comfortable in playing a role as a PrEP peer educator.

*“I think the best way to help someone like me to promote this would be to give me the information. If I had all the information that I’m curious about*,* it’d be a lot easier for me to spread the message of what PrEP is and why it’s important…For me*,* again*,* like I mentioned*,* I’m all about the facts. I need to know the science behind it. I need to know the truth. I don’t wanna know your opinion*,* how you feel about it. Show me the paperwork. If it’s on paper*,* it’s good to go for me.”* - *White/Hispanic male*,* mid-thirties*.

Since many participants noted that cost was a major barrier to PrEP uptake, some suggested including information about how to pay for PrEP in the intervention, including available financial assistance programs.

*“The training that I think I would need is I would want to have more information on the how it can be affected by insurance*,* ‘cause that’s another big question*,* is how can people afford it? I’m not gonna lie*,* I know about my personal insurance*,* but I know everyone’s different and there’s different things*,* we’ve got different tiers. I think that that’s a big one*,* because that’s gonna be a question*,* I feel like*,* everyone’s gonna ask is*,* ‘Financially*,* is it realistic for me to do this?’” – Black/non-Hispanic male*,* late-twenties*.

### Approaches to initiate PrEP conversation

We asked participants about how best to initiate a conversation about PrEP with peers in their social network. A range of approaches were identified such as: letting the conversation occur naturally, asking their peers directly if they have heard about PrEP and then providing more information if they have not, sharing personal experiences related to PrEP, providing an informational sheet about PrEP without first discussing it at-length, and only providing information if someone asks about it first.

*“I think it just comes up casually*,* I don’t think that there is an intended approach. If I’m talking to my friends—for example*,* yesterday…my friends asked*,* “Hey*,* so what are you up to today?” I said*,* “Well*,* I have my PrEP appointment at this time.” Then*,* the conversation organically flows from that…I don’t go out of my way to say*,* “Wait*,* what about PrEP?” It doesn’t come like that. It’s usually more like a casual conversation.” – Hispanic male*,* mid-thirties*.

*“I would probably just ask them ‘have they heard of it.’ Then*,* from there*,* gauge what they knew of PrEP. Then*,* probably in that*,* hear any concerns they would have*,* and just try to use my experience to help them gain access to it.”* - *Black/non-Hispanic male*,* early-thirties*.

*“As opposed to having a long conversation [with someone you don’t know]*,* when you don’t know if someone’s interested in that conversation*,* you can very easily give them a card and just say*,* “Think about this.” I think it’s a good way to do it.” – Black/non-Hispanic male*,* early-twenties*.

Participants identified text messaging, social media, and in-person meetings as preferred methods to deliver a PrEP PDI. Some suggested text messaging as an easier and more convenient delivery method of information than meeting in-person and identified social media as being able to reach a large audience quickly.

*“I think*,* in this day and age*,* social media and texting is probably the best way to get in contact with people. That’s definitely how I would approach it*,* just a friendly little message in text or social media.”* - *Black/non-Hispanic male*, *early-thirties*.

*“Maybe a social network would be a really good way to spread that message because you can spread one thing—you can share one thing on*,* let’s say Facebook. Within one hours that one message you’ve just shared could reach the other side of the world. Hundreds and thousands of people on the other side of the world.” – White/Hispanic male*,* mid-thirties*.

However, some participants demonstrated a strong preference for in-person approaches, stating they believed it would be more effective in establishing a connection.

*“The best way would be in-person. I think all the best conversations are face-to-face conversations.” - White/non-Hispanic male*,* late-seventies*.

*“I’m old fashioned. I do the in-person just ‘cause I think that does capture everything. Sometimes via text or calling*,* you do lose some of that situational reaction. You lose a lot*,* you lose facial expression*,* you lose a lot of different things that can eventually help an individual make the right step in their life.” – White/Hispanic male*,* mid-twenties*.

### Cultural beliefs impact on PrEP uptake

Participants generally agreed that cultural beliefs may act as barriers to initiating PrEP. For example, some cultures do not accept same-sex relationships, and therefore those who belong to these cultures may find it uncomfortable to talk about sexual health and be unable to pursue PrEP.

*“I definitely think there are some cultures that are not open to same-sex relationships or sex before marriage and things like that. That shame and stuff*,* I’m sure*,* can trickle in for individuals when it comes to taking something like PrEP.”* - *Black/non-Hispanic male*,* early-thirties*.

One participant who identified as B/AA discussed how internalized stigma and low self-esteem, resulting from structural racism within our society, can act as a barrier to initiating PrEP. In addition, according to this participant there may be a misconception among some B/AA MSM that PrEP is not as applicable to them as it is for White MSM.

*“I think we live in a culture that still is reckoning with racism. I think one of the effects of that is that if someone has a hard time seeing themselves as valuable or worthy of being treated well and being healthy*,* then they’re also less likely to practice—have good sex practices. That strikes me as a pretty big cultural barrier. …I also think*,* just in terms of how segregated people are*,* I think there is still an image of PrEP as something that mostly white gay men do. I think that’s a piece of it too.”* - *Black/non-Hispanic male*,* early-thirties*.

Some H/L participants shared their experiences on being from a community where conversations about sexual health may not be normalized, and how that creates a barrier to initiating PrEP.

*“I think sex sometimes is one of those where*,* in a lot of Hispanic countries*,* or not countries itself*,* but little communities…You don’t talk about sex at a dinner table. You don’t talk about sex with your friends. You only talk about sex with your spouse*,* or whatever the case is. In some cases*,* your parents won’t even talk to you about sex because it’s taboo*,* and you shouldn’t. They don’t even refer to genitalia as penis and vagina. They just go*,* like—they call it by other cutesy names*,* because they’re embarrassed to talk about sex. I think*,* culturally*,* there is a lot of barriers*,* and I think that could be a struggle when it comes to PrEP knowledge. Because a lot of—whether it’s a Hispanic gay man*,* or a Hispanic straight male*,* they may not be willing to talk about it. They even may think they don’t need it.”- Hispanic male*,* mid-thirties*.

### Unique barriers for young adults interested in PrEP

One young adult participant mentioned that being on their parents’ insurance was a key barrier to initiating PrEP. They started to take PrEP only once they were on their own health plan.

*“I wanted to make sure that I was on my own insurance*,* ‘cause I’m not out to my parents. I don’t think we had that insurance where I would get on somethin’ and they would notify them*,* but I’m a very private person when it comes to a lot of things in my life. That was one of the main factors for me [to start PrEP]*,* that I was off my parent’s insurance and I had my own insurance and I was able to afford it.”- Black/Hispanic male*,* early-twenties*.

Another young adult, recently graduated from college, reported a lack of comprehensive sexual health education and PrEP resources available through their school.

*I think a lot of [sexual health education] came from*,* honestly*,* off-campus resources*,* once I started going to a clinic outside of my college campus….my college campus was the one who recommended [PrEP] for me*,* but they told me to go off campus to supply it. – White/non-Hispanic male*,* early twenties*.

### The impact of COVID on PrEP use and access

All participants reported that the COVID-19 pandemic drastically changed the way they socialize and communicate, and their sexual activity. During the COVID-19 pandemic, most individuals had minimal in-person social activities and reduced sexual activity as well.

*“Pre-COVID…I had more sexual partners for sure. Now*,* during COVID*,* if I chat with someone—you add the question on top of like*,* “Hey*,* what’s your status?” …You’re not necessarily just asking these days for what’s your HIV status*,* you’re also asking what’s your COVID status? Are you negative? Are you safe? Some people will just give you different answers. I have chosen not to engage in too many sexual encounters in 2020.”- Hispanic male*,* mid-thirties*.

Due to reduced sexual activity and lower perceived risk of HIV acquisition during the pandemic, some participants chose to temporarily discontinue PrEP.

*“At the beginning of the pandemic*,* I had stopped taking it*,* ’cause there was no need. I wasn’t having any sexual activity for a few months…Once restriction and things were loosening up a bit*,* that’s when I was being sexually active again. That’s when I started [PrEP] back up.” –Black/non-Hispanic male*,* early-thirties*.

The COVID-19 pandemic often negatively impacted participants’ access to medical care, including PrEP appointments and routine testing.

*“There was interruptions…at the height of COVID*,* so the very early stages where a lot of medical or health organizations or providers were going teletherapy. You were able to do your consulting or your check-in appointment*,* but unless you had symptoms of something*,* they were like*,* “Okay*,* don’t go to the doctor. Don’t do the three-month blood work of whether it’s your kidney or your STD bloodwork*,*” or whatever. It became very like*,* okay…so that’s kinda why I got off of it.”- White/Hispanic male*,* mid-thirties*.

Several participants reported having prescription refills delivered at-home to mitigate COVID-related barriers to PrEP access.

*“The pills usually get delivered to my…so it’s usually pretty straight forward. I’m home all the time*,* so I can’t really miss a dose.” – Hispanic male*,* mid-thirties*.

However, a couple participants who received their PrEP refills via at-home delivery reported major issues with the delivery service that negatively affected their adherence to PrEP.

*“There was two weeks where I just wasn’t getting it delivered. I called the people and said*,* “Hey*,* I signed up for delivery. Why haven’t you sent it?” They eventually just signed me up for delivery permanently. Now I get it delivered automatically*,* which is good…I did have a two-week gap that one time. Since then*,* I’ve been covered.” – Black/non-Hispanic male*,* early-twenties*.

*“The process in which I can reach and get [PrEP] is a bit hard. Because they are not found in the nearby stores*,* I need to order online and delivery sometimes is delayed*,* so that’s a big challenge to me.” – Black/non-Hispanic male*,* late-twenties*.

## Discussion

This study is one of the first to explore the acceptability and potential components of a PDI for PrEP among MSM. Our findings highlighted a positive perception of a PDI for PrEP among participants, with a willingness to both educate and learn from their peers. This is consistent with previous studies that have explored the effectiveness of PDIs in promoting various HIV prevention measures, such as adherence to antiretroviral therapy (ART) and increased condom use, particularly within populations disproportionately affected by HIV [27, 37–39]. These findings are also consistent with the pilot study conducted by the authors on a PDI for PrEP uptake among MSM [30]. This current study had nearly double the participants of the pilot, with no participants that were involved in both studies, and was conducted during and post-COVID, which has allowed us to capture a broader range of perspectives and analyze how the COVID-19 pandemic has changed our study population’s social interactions, sexual behaviors, and PrEP access. Given the alignment of our study’s findings with existing literature and the urgent need for PrEP promotion, especially among B/AA and H/L MSM who face a disproportionate burden of HIV infection, a PDI approach holds significant promise to reducing this burden among the most affected populations [25, 29]. Our study suggests that PDIs have the potential to address disparities in PrEP uptake and contribute to reducing HIV transmission within these communities.

We conducted an in-depth exploration of the essential components necessary for an effective implementation of a PDI to promote uptake of PrEP. This exploration encompassed various topics, including shared characteristics of social networks among MSM, the role of peers in raising PrEP awareness, general attitudes toward a PDI approach, desired attributes and formal trainings of peer educators, approaches to initiate conversations about PrEP, and cultural barriers to PrEP initiation. Through identifying and investigating these themes, our results have important implications for understanding the various elements that should be in place for a PDI tailored to PrEP promotion to succeed.

Most study participants reported having a diverse group of friends with varied racial and ethnic backgrounds. They also mentioned having a close-knit circle of friends who identified as MSM. This diverse network facilitated open and comfortable discussions about sex, HIV, and drug-related topics among their social connections. Participants’ ability to engage in such conversations was influenced by characteristics of their social network, and if they had access to an environment where important health-related discussions could take place without fear of judgment or stigma. However, it’s important to recognize that this sample may not reflect the wider Black and Hispanic MSM populations, even in our study setting. Caution is warranted when extrapolating that such supportive discussions are uniformly prevalent in diverse networks across these communities. Additionally, as indicated by responses in the study, many Hispanic and Black communities face significant sex- and racism-related stigma. This stigma poses a substantial, yet not unassailable, obstacle to the effectiveness of interventions. The findings underscore the need for nuanced approaches to health promotion that consider the complex interplay of cultural, social, and individual factors. Our participants highlighted the importance of selecting peer educators who are outgoing, personable, and skilled in effective communication. Moreover, peer educators should exhibit a strong knowledge base and enthusiasm for PrEP and be willing to educate individuals within their own social circles. Furthermore, peer educators who share experiences or backgrounds with participants were seen as having an advantage in building trust and rapport. This aligns with existing research demonstrating that individuals at the center of social networks exert greater influence over the health behavior of others within their network [40].

Participants identified several critical components to be included in PrEP education content. They stressed the need to emphasize the higher HIV risk among MSM compared to heterosexuals and suggested presenting current HIV data during sessions to underscore this. They also wanted to see evidence from previous studies demonstrating PrEP’s effectiveness in HIV prevention. Additionally, they emphasized the importance of discussing adherence to maximize PrEP’s effectiveness and desired more information on how PrEP functions in the body, available PrEP options, and candid discussions about potential side effects. Lastly, many participants suggested that it would be beneficial for peers providing PrEP education to include information about financial assistance programs for PrEP and how to navigate insurance when initiating PrEP. Given that neither of the two uninsured participants were currently on PrEP, it is likely that the existence of PrEP financial assistance programs for uninsured individuals is not widespread knowledge. Therefore, to fill this gap in PrEP coverage of uninsured patients, future peer-based interventions should include information about these programs and assist patients in applying for them.

It is crucial that PDI approaches include culturally appropriate content and address the cultural barriers mentioned by B/A and H/L participants. Given that several participants voiced they would be more comfortable with a peer educator with whom they shared an identity or cultural background, it may be highly beneficial for a PrEP PDI to select peer educators who are B/AA or H/L MSM currently on PrEP or with prior PrEP experience. Training for peer educators should also include how to approach conversations about PrEP with a focus on navigating cultural barriers that may inhibit sexual health conversations as well as the effects of internalized stigma. Such cultural competence will ensure that the intervention resonates with the targeted population.

Many participants suggested and preferred in-person meetings for PDIs. Some participants expressed a strong preference for face-to-face interactions, emphasizing the efficacy of personal engagement in providing information and addressing concerns. Conversely, other participants exhibited a greater openness to digital modes of communication. Nevertheless, it’s worth noting that the evolving landscape of social interactions influenced by the COVID-19 pandemic has prompted a shift in preferences, with some participants preferring virtual modes of engagement.

We also found that young adults face unique barriers when considering PrEP initiation, as highlighted by some participants in our study. For one individual, a key obstacle was enrollment on their parent’s insurance plan. For young adults still on a parental insurance plan, navigating sensitive conversations with their parents regarding their sexual health choices can pose a daunting challenge, in particular for those with parents unaware or unsupportive of their sexual orientation. Another participant disclosed that their university health services were not equipped to prescribe or provide PrEP, and that sexual health education and resources through their university were lacking. These experiences underscore the need for tailored interventions and support systems to address the unique concerns of young adults, including college students, interested in PrEP.

The COVID-19 pandemic caused significant changes in participants’ social and sexual behaviors. Lockdowns and social distancing measures led to reduced in-person social activities and fewer sexual encounters. Consequently, some participants temporarily stopped taking PrEP as they perceived their risk of HIV acquisition to be low. The pandemic posed challenges to consistent PrEP access and adherence due to disruptions in healthcare services and personal circumstances. Fear of COVID-19 infection also deterred participants from visiting healthcare facilities for PrEP prescriptions. Despite efforts like at-home medication delivery, some participants faced issues that hindered their adherence. HIV prevention initiatives should enhance PrEP accessibility through measures such as affordability without insurance and improved delivery options.

In our study, we employed purposive sampling based on PrEP status and race/ethnicity to explore attitudes towards a PDI on PrEP promotion among individuals with varying PrEP experiences and racial/ethnic backgrounds. This sampling methodology may have influenced our observation that B/AA MSM had a higher likelihood of PrEP usage in this study, a finding that diverges from the predominant trends reported in existing literature [41]. It is reasonable to consider that B/AA MSM who are currently taking PrEP could be more motivated to participate in a study focusing on PrEP-related interventions. Given that the primary aim of our study was to explore knowledge of and perspectives on PrEP, this potential sampling bias is unlikely to significantly detract from the validity of our findings. Indeed, capturing the insights of active PrEP users is integral to understanding the nuances of PrEP promotion strategies within diverse communities.

Nonetheless, it is important to consider the limitations of this study. The insights presented here are based on interviews conducted in the state of Rhode Island and variations in perspectives may exist in different geographical and cultural settings. While we employed purposive sampling to capture diverse viewpoints, the findings may not fully represent the broader MSM population, especially B/AA and H/L MSM. Additionally, the study was conducted during the COVID-19 pandemic, which may have impacted preferences for in-person meetings and social interactions. Aware of the potential for social desirability bias, we employed a non-judgmental and supportive interview approach, reassuring participants that all responses were valid without any ‘correct’ answers, to mitigate this effect.

## Conclusions

In conclusion, this study suggests ways to develop a tailored PDI approach for promoting PrEP uptake among MSM populations, in particular B/AA and H/L MSM. Our findings suggest that the success of PDIs will be feasible and acceptable but their success will depend on careful selection of peer educators, comprehensive training, culturally-sensitive content, and the acknowledgment of unique challenges related to HIV and PrEP within these communities.

## Electronic supplementary material

Below is the link to the electronic supplementary material.

Supplementary Material 1

## Data Availability

The datasets generated and/or analyzed during the current study are not publicly available due to risk of compromising privacy of the qualitative interview participants but are available from the corresponding author on reasonable request.

## Abbreviations

PrEP: Pre-exposure prophylaxis
PDI: Peer-driven intervention
B/AA: Black/African American
H/L: Hispanic/Latino
MSM: Men who have sex with men
CDC: Centers for Disease Control and Prevention
HIV: Human immunodeficiency virus
LGBTQ: Lesbian, gay, bisexual, transgender, queer
