# Supplementary material for: Perspectives on a peer-driven intervention to promote pre-exposure prophylaxis (PrEP) uptake among men who have sex with men in southern New England: a qualitative study
Source: BMC Health Serv Res. 2024 Sep 4;24:1023. doi: 10.1186/s12913-024-11461-7 (PMC11376045; doi:10.1186/s12913-024-11461-7)
Supplement: Supplementary file 1 — Supplementary Material 1 [file 12913_2024_11461_MOESM1_ESM.docx]

RIN: ________ Today's date: __________

Start time: ____________

**Interview Guide**

**Peer-driven intervention prompting pre-exposure prophylaxis and improving access to health care among African American and Hispanic/Latino men who have sex with men**

Overview: The purpose of this interview guide is to gather information on the acceptability of a peer-driven intervention (PDI) to promote pre-exposure prophylaxis (PrEP), components of an effective PDI, and barriers to PrEP uptake. The PDI approach will use index peers to disseminate knowledge of HIV and PrEP and encourage members of their social networks to use PrEP. This document is intended to guide interviewers through the key content areas of data collection for this project, ensuring that the same content is discussed with each participant. While the interview guide is used to facilitate the discussion, it is not a rigid script that must be adhered to verbatim. This ensures that the interviewer gathers data on the same topics with each participant, while also allowing the flexibility to adapt and clarify questions to suit the needs of different individuals. Similarly, questions need not be asked in this particular order. The facilitator will adapt the conversation for each participant, pursuing both the *a priori* research topics as well as any emergent relevant themes that evolve during the interview.

**Welcome procedures**

Interviewer Instructions: Welcome participant, explain basic study details and purpose, explain that interviews are being audio-recorded, and discuss how all information will be kept confidential. In addition, ask participant to turn off their cell phone.

**Introduction**

Interviewer: *Welcome and thank you for agreeing to be a part of this interview. I will ask you a series of questions about a pill some people take once a day to prevent HIV (known as once-daily oral PrEP for short), your initial thoughts about using people you may encounter in your daily life (peers) to promote PrEP use, and your suggestions about components of a promotion program led by people you know. There are no “right” or “wrong” answers to my questions. Rather, I’m interested in any information that you can provide based on your own personal experiences, observations, or feelings.*

*Please be assured that all of your responses are confidential. Our summary report and publications of the information we collect from you will make no reference to your name or other identifying information. As a reminder, this interview will be audio-recorded to be sure that we do not miss any information that you share with us. We ask that you please turn off your cell now and keep it off for the remainder of the interview.*

*Your candid responses and discussion will be most helpful to us as we look to design an approach led by people you know to promote PrEP use and other related issues.*

*Before we begin, what questions or concerns do you have?*

Interviewer Instructions: For participants on PrEP, complete sections 1-4 and 6-8. For participants not on PrEP, complete sections 5-8.

| **Demographics**  *To be administered by interviewer* | |
| --- | --- |
| **A1. What is your age?** | _______ years |
| **A2. What is your gender?** | ___ Woman  ___ Man  ___ Trans woman  ___ Trans man  ___ Genderqueer/gender fluid/nonbinary  ___ Agender  ___ None of these *(please write in)*: ______________ |
| **A3. What was your sex assigned at birth?** | ___ Female  ___ Male  ___ Intersex/another sex |
| **A4. Which of these best describes your sexual orientation?** | *____*Heterosexual or straight  ____Bisexual  ____Gay, lesbian, or same gender loving  ____Not sure or questioning  ____None of these *(please write in)*: ______________ |
| **A5. What is your race?** | *____*White  *____*Black or African American  *____*American Indian or Alaska Native  *____*Native Hawaiian or other Pacific Islander  *____*Asian  ____None of these *(please write in)*: ______________ |
| **A6. Do you consider yourself Spanish, Hispanic, or Latino?** | ____No  ____Yes - Mexican/Mexican American/Chicano  ____Yes - Puerto Rican  ____Yes - Cuban  ____Yes - Dominican  ____Yes - Guatemalan  ____Yes - Other ethnicity: ______________ |
| **A7. What is your relationship status?** | ___ Single  ___ Monogamous relationship (one partner)  ___ Non-monogamous (multiple partners – polyamorous,  open, etc.) relationship  ___ None of these *(please write in)*: ______________ |
| **A7i. IF APPLICABLE: How long have you been in a relationship with your CURRENT main partner?** | *Please leave spaces blank if they do not apply to you.*  ______ (weeks) (months) (years) |
| **A8. What is your highest completed level of education?** | ___High school or less  ___Some college  ___College  ___Graduate school |
| **A9. Are you currently a student?**  (This includes in a school or program working toward a high school diploma, GED, or college/technical degree) | ___Yes  ___No |
| **A10. What is your employment status?** | ___ Full-time  ___ Part-time  ___ Unemployed  ___ Retired  ___ Disability  ___ Other: _______ |
| **A11. What is your annual income?** | ___ Less than $10,000  ___ $10,000 to $15,000  ___ $15,000 to $20,000  ___ $20,000 to $25,000  ___ $25,000 or 35,000  ___ $35,000 or 50,000  ___ $50,000 or 75,000  ___ $75,000 or more |
| **A12. Do you have health insurance?** | ___ No  ___ ___Yes - private insurance (e.g. Blue Cross/Blue Shield, United Health, Aetna, Cigna, Tufts)  ___ ___Yes – Medicaid (e.g., Neighborhood Health Plan, United Health Ritecare, or MassHealth)  ___ Yes - Medicare  ___ Yes - military/veterans insurance  ___ Yes - Hospital Free Care Program  ___ Yes - other insurance: ______________ |
| **A13. Have you had unstable housing in the past six (6) months?**  (“Unstable housing” means living in a hotel, boarding house, group home, in the street, or having no fixed address) | ___Yes  ___No |
| **A14. Have you ever spent time in jail or prison, even if you were only held a short time?** | ___Yes  ___No |
| **A15. Have you heard of PrEP (pre-exposure prophylaxis), a daily pill to prevent HIV?** | ___Yes  ___No |
| **A15i. *IF YES*: Have you ever been prescribed a daily pill for PrEP?** | ___Yes  ___No |
| **A15ii. *IF YES – PRESCRIBED:* What is the name of the doctor or clinic where you received a PrEP prescription?**  *(This question is for analytic purposes only – we will not contact this doctor or clinic about your survey responses.)* | Name: ________________________________________  City, State: ____________________________________ |
| **A15iii. *IF YES - PRESCRIBED*: Are you currently taking a pill for PrEP?** | ___Yes, I try to take it every day  *___*Yes, but I only take it when I feel I need it  ___No  ___Unsure |

**General Content Areas**

1. **Experiences with Oral PrEP (TAKING Oral PrEP) and perspectives about PrEP promotion by people you know**
   1. Why did you start taking once-daily oral PrEP?
      1. What was the main reason you started taking PrEP?
      2. What (if any) challenges did you initially encounter to taking PrEP?
      3. What made it easier to initially start taking PrEP?
      4. Did you worry about what people would think once you started?
   2. How long have you been taking PrEP?

i. If you initiated PrEP during the COVID-19 pandemic, what’s your experience been like?

How did your sexual behaviors change during COVID-19? How did this affect your PrEP use?

- 1. What were your initial thoughts about taking a prescribed daily oral medication to reduce your risk of getting or becoming infected with HIV?
  2. Tell me about your experience taking PrEP.
  3. Prior to taking PrEP in this clinic, had you heard of PrEP? Where did you first hear about it?
  4. Have you talked with people you know about PrEP?
     1. If yes, how did you initiate this topic?
     2. If no, why not? What are the main concerns?
  5. How many other gay or bisexual men do you know? Did someone you know take PrEP?
     1. If yes, do you know their experience with PrEP?
     2. If no, what are main barriers to PrEP uptake for them? Do you think you are able to address some of their concerns?
        1. Low risk of HIV infection;
        2. Not knowing enough about the medication or how to take it;
        3. Not wanting to take a medication every day;
        4. Remembering to take a medication every day;
        5. What other people would think of them;
        6. People would think they are HIV positive;
        7. The cost of the medication (co-pay, deductible, co-insurance);
        8. The cost of going to the doctor (co-pay, deductible, co-insurance);
        9. Transportation (to the doctor office or pharmacy);
        10. Work schedule;
        11. Using drugs;
        12. Drinking alcohol;
        13. What other barriers might keep members of your social network from starting or staying on oral PrEP?
  6. What about your attitude to disseminate knowledge of PrEP and encourage people you know to take PrEP?
  7. If you are willing to promote PrEP in your community, what approaches will you use?
     1. Call people
     2. Text people
     3. Social media (which platform would be best? e.g. Twitter, Facebook, Instagram)
     4. Online hook-up apps (which app would be best? E.g. Grindr, scruff, multiple, etc.)
     5. Community events (which ones?)
     6. School events
     7. Other (e.g. church, etc.)

How would you determine if someone could benefit from PrEP? Tell me about an example of a person you actually know, why you think they may benefit from PrEP, and how you would consider bringing it up to them and what you would say.

We are considering offering to train people about how to talk to other people about PrEP and refer them to us for PrEP care. Do you think people would be willing to be part of this program? Why or why not? What should people know about PrEP?

What topic(s) would you need to know more about before training others about PrEP?

- - 1. Knowledge of HIV
    2. Knowledge of PrEP
    3. Communication skills (e.g. eye contact, body language)
    4. Anything else

What do you think would be the biggest barriers to a program such as this? Probe cultural barriers.

We are considering offering some financial incentives to the person who is referring others for PrEP. This would cover their time for the training and would be based on how many people they refer in. What amount per person would you think is reasonable?

We are also considering offering financial incentives to people who are referred for PrEP to encourage them to attend an appointment. What amount would you think is reasonable?

1. **Adherence to Daily Dosing (TAKING Oral PrEP) and perspectives about PrEP promotion by people you know**
   1. Are you taking any other medications?
      1. If yes: How, if at all, did adding once-daily oral PrEP to your medications affect how you take your medications?
   2. Do you take once-daily oral PrEP at the same time you take other medications?
      1. With food or anything else?
   3. Has COVID-19 led to any barriers in terms of taking your PrEP medication every day?
      1. Were you able to attend clinical visits to see your medical provider for PrEP? Were these in-person or telemedicine?
      2. If telemedicine, how did that go? What did you like or dislike about it?
      3. Any issues with refilling medication?
      4. Any other barriers?
   4. About what percentage of the time would you say you take your medications every day?
      1. Oral PrEP?
      2. Other medications?
   5. What time of day do you usually take your medications?
   6. How do you remember to take your medications? If you ever miss taking your medications, what is the main reason?
   7. Have you ever taken oral PrEP intermittently, just every once in a while?
      1. If so, why?
      2. Some people who take PrEP intermittently only take PrEP around times when they have sex. How did you decide when to take PrEP?
   8. We all forget to take our medications at times. Tell me about a time you forgot to take your PrEP medication. Probes:
      1. Is there anything that makes it hard for you to take PrEP?
      2. What do you do when you forget to take PrEP?
      3. Who reminds you to take PrEP?
   9. We are considering using index peers to remind of taking their medications each day. How would you feel about that?
      1. What time of day would you prefer to be reached?
      2. Which method would you prefer to be reached?
      3. What would you like to be included in reminders?
      4. What else could we do to help you take your medications each day?
   10. Are you willing to share any suggestions you have to help people you know to remember to take their medications?
       1. If yes, what approaches will you prefer? What concerns may you have?
       2. If no, why?
   11. What trainings do you need to facilitate reminder of taking their medications?
2. **Side Effects (TAKING Oral PrEP) and perspectives about PrEP promotion by people you know**
   1. Have you had any side effects associated with taking once-daily oral PrEP, especially around the time that you started taking it?
      1. Nausea? Diarrhea? Anything else? Tell me about that.
   2. Are you currently experiencing any problems associated with taking your once-daily oral PrEP?
   3. Did you discuss those problems with your doctor?
   4. Did the side effects ever prompt you to stop taking your medications? Tell me about that.
   5. When you are taking PrEP, your provider should be testing you for HIV and other STDs routinely. Please tell us about your experience with frequent testing.
   6. What’s your opinion about the increased medical visits and testing that come with taking PrEP burdensome or annoying?
   7. What’s your opinion about support from people you know could ease side effects of PrEP?
      1. If yes, could you please talk about how it can work?
      2. If no, why?
   8. Are you willing to help people who suffer from side effects of PrEP in your community?
      1. If yes, what are your approaches?
      2. If no, why?
   9. What training that you may need to help them?
3. **Sexual Risk Behaviors (TAKING ORAL PrEP)**
   1. Thinking back to the time before you started taking once-daily oral PrEP, did you feel you were at risk for contracting HIV? Can you tell me a little bit about your sex life before you started taking PrEP? Were you sexually active when you started taking PrEP?
      1. Number of partners
      2. Sex of partners (men, women, transgender, not transgender)
      3. Concurrent partners (i.e., overlapping, or more than one, sexual partners during a certain time period)
      4. Condom use with main and non-main partners
      5. HIV-positive partner or partners of unknown status
      6. Vaginal, oral, or anal intercourse? *If anal:* Do you usually top, bottom, or do both?
   2. Now that you’re on once-daily oral PrEP, tell me a little bit about your sex life. Are you currently sexually active? Is there anything you’re doing differently now that you’ve been on PrEP?
      1. Number of partners
      2. Sex of partners (men, women, transgender, not transgender)
      3. Concurrent partners
      4. Condom use with main and non-main partners
      5. HIV-positive partner or partners of unknown status?
      6. Vaginal, oral, or anal (insertive/receptive) sex?
      7. Where do you usually meet your partners?
   3. Can you tell me a little bit about your sex life pre- and post- COVID-19?
      1. Number of partners
      2. Sex of partners (men, women, transgender, not transgender)
      3. Concurrent partners (i.e., overlapping, or more than one, sexual partners during a certain time period)
      4. Condom use with main and non-main partners
      5. HIV-positive partner or partners of unknown status?
      6. Vaginal, oral, or anal (insertive/receptive) sex?
      7. Where do you usually meet your partners? How did this change during the COVID-19 pandemic?
   4. Some people believe that people taking PrEP may stop using condoms, or have more sexual partners because they think they are no longer at risk for contracting HIV. What do you think about that?
      1. Has taking PrEP caused you to use condoms less frequently?
      2. To have more sexual partners?
      3. To think you are no longer at risk for contracting HIV?
   5. Tell me how taking PrEP might impact your decision to get tested for HIV or other STDs in the future.
   6. Tell me about whether and how taking PrEP might affect your sexual decisions in the future.
   7. Have you changed your primary sex position based upon being on PrEP? For example, do you feel more comfortable now being the receptive partner (“bottom”) versus the insertive partner (“top”), or vice versa?
4. **Initial Feelings about PrEP (NOT taking PrEP) and perspectives about PrEP promotion by people you know**
   1. How familiar are you with PrEP?
   2. Prior to today, what had you heard about PrEP?
   3. What are your initial thoughts about taking a prescribed daily oral medication to reduce your risk of contracting HIV?
   4. Have you considered taking once-daily oral PrEP?
      1. If so, what has prevented you from doing so?
      2. If not, why?
      3. Would any of the following be a barrier to starting or staying on oral PrEP:
         1. I don’t think I am at risk of HIV infection;
         2. Not knowing enough about the medication or how to take it;
         3. Not wanting to take a medication every day;
         4. Remembering to take a medication every day;
         5. What other people would think of me;
         6. People would think I am HIV positive;
         7. The cost of the medication (co-pay, deductible, co-insurance);
         8. The cost of going to the doctor (co-pay, deductible, co-insurance);
         9. Transportation (to the doctor office or pharmacy);
         10. Work schedule;
         11. Using drugs;
         12. Drinking alcohol;
         13. What other barriers might keep you from starting or staying on oral PrEP?
   5. How did the COVID-19 pandemic impact your decision about taking PrEP?
   6. Would you like to be approached by people you know or providers about PrEP?
      1. If you prefer people you know, why?
      2. If you prefer providers, why?
   7. Is someone you know taking PrEP currently?
      1. If yes, would you like to be approached by PrEP users in your community about PrEP use?
      2. If no, why?
   8. What approach methods should people you know use for PrEP promotion?
   9. What characteristics should a PrEP promoter have?
   10. What contents are you willing to hear about?
   11. How likely will it be for you to be motivated by people you know?
   12. Are you willing to disseminate knowledge of PrEP, and encourage people you know to take PrEP?
   13. What do you think is your risk for contracting an STD pre- and post-COVID-19? Why?
   14. What do you think is your risk for contracting HIV pre- and post-COVID-19? Why?
       1. Can you tell me a little bit about your sex life pre- and post- COVID-19?Number of partners
       2. Sex of partners (men, women, transgender, not transgender)
       3. Concurrent partners (i.e., overlapping, or more than one, sexual partners during a certain time period)
       4. Condom use with main and non-main partners
       5. HIV-positive partner or partners of unknown status
       6. Vaginal, oral, or anal intercourse? *If anal:* Do you usually top, bottom, or do both?
       7. Where do you usually meet your partners?
       8. Have you ever had an STD?
          1. Which one(s)? When?
       9. Have you ever injected drugs or hormones with a needle?
          1. If so, have you ever shared needles?
          2. When was the last time? What drugs?
5. **Attitudes about healthcare (ALL participants) Now I’m going to ask you about your experiences with health care providers and the health care system in general.**
   1. Tell me about how comfortable or uncomfortable your past experiences with health care providers (e.g., doctors, nurses) and clinics have made you feel.
      1. How comfortable or uncomfortable are you with telling your providers about your health and sexual behaviors?
      2. What role do you think your racial or ethnic identity has played in the way your providers have treated you in the past? Have you ever felt discriminated against by providers because of your racial or ethnic identity?
      3. What role do you think your sexual orientation/sexual identity has played in the way providers have treated you in the past? Have you ever felt discriminated against by providers because of your sexual orientation/sexual identity?
      4. How do you think past experiences with providers may have impacted your health care decision-making, such as getting tested for HIV, taking PrEP, or any other care?
      5. What’s your experience pre- and post-COVID-19?
   2. Think about your interactions with the healthcare system overall—this includes doctors and other health care providers, clinics, health insurance companies, and laws and policies. How much or how little do you trust the healthcare system?
      1. How much do you trust that the healthcare system acts in your best interest?
      2. How often do you avoid or put off seeking healthcare because you do not feel comfortable or do not trust the system?
      3. How have HIV/AIDS conspiracy theories (beliefs about government involvement in the creation of HIV, to beliefs that testing and HIV medications themselves can be used as instruments to wipe out “undesirable” populations) served as obstacles to HIV prevention including HIV testing and use of PrEP for you and/or your community?
      4. How have language or other cultural barriers served as obstacles to HIV prevention including HIV testing and use of PrEP for you and/or your community?
6. **Social Network**

Now, I would like to know a little about the people you normally hang out with.

- 1. Tell me about the people you spend the most time with.
  2. Who are they? (Close friends? Acquaintances? Co-workers? Family members? Sexual partners?)
  3. What things do you and your friends normally do together pre- and post-COVID-19?
  4. Where do you typically hang out and why pre- and post-COVID-19? What do you normally do on a day off pre- and post-COVID-19?
  5. Where do you usually find sex partners (e.g. Grinder, Jack’D, and gay clubs) pre- and post-COVID-19?
  6. Who do you feel are the most important or influential people you know? What makes them so important or influential to you?
  7. How do these people influence you? How do you influence them?
  8. How do you communicate with the people who are most important to you pre- and post-COVID-19? What do you talk/communicate about most?
  9. How do you talk about sex? HIV? Drugs?

1. **Other (ALL participants)**
   1. Tell me about your overall experience at our clinic.
   2. Did you feel that we explained the risks and benefits of taking once-daily oral PrEP to your satisfaction?
   3. Is there anything we could do to improve your experience with STD testing and treatment or once-daily oral PrEP here?
   4. Is there anything else you’d like to share about your experience with HIV/STD testing at our clinic?
   5. *If taking PrEP:* How long do you think you will continue to take once-daily oral PrEP?
   6. We want to improve the patient experience with PrEP education. I appreciate your time and input. Is there anything else you would like to tell me that I haven’t asked you?
   7. Is there anything else that I have not already asked you about during the entire interview that you would like to share with me?

**Closing procedures**

Interviewer Instructions: Thank participant for their assistance today. Remind them that all of the information they shared will be kept confidential. Give participant reimbursement and record the time when interview was completed.

Interviewer: *Thank you again for your participation today. We really appreciate your assistance. As a reminder, everything that you shared with me today will be kept confidential*.

*What final questions or concerns do you have for me*?

*OK, great. Thank you again and here is your reimbursement. Have a nice day!*

Finish time*: ___________*

Suggestions:

1. Please block 30 minutes before each interview session. You will use this reserved 30 minutes to go over the interview guide and plan how to lead the conversation.
2. You can go off the scripts and tell more about our peer-driven intervention study.

Example: we plan to run a peer-led intervention to spread HIV/PrEP knowledge to your communities, we would like to hear your opinions to this approach and how can we make it work. Really appreciate you are willing to share your experience with us.

1. Please try to have a short introduction first and let the participant introduce himself. It will be a good way to warm up and let the participant feel relax and comfortable.
2. You can make notes during the interview. Please pay attention to their words. Do not ask questions when you already got answers during the conversation. You could rephrase what you heard and check with participants. They will feel you are engaged in the conversation.
3. It is always great to appreciate they share their information. But, it is very important to probe.

*Just like the case yesterday, when he said he had a low risk to get HIV. You should ask why.*

1. Only aske one question at a time.
